# Escitalopram Decreases Cross-Regional Functional Connectivity within the Default-Mode Network

**DOI:** 10.1371/journal.pone.0068355

**Published:** 2013-06-27

**Authors:** Vincent van de Ven, Marleen Wingen, Kim P. C. Kuypers, Johannes G. Ramaekers, Elia Formisano

**Affiliations:** 1 Department of Cognitive Neuroscience, Faculty of Psychology and Neuroscience, Maastricht University, Maastricht, The Netherlands; 2 Department of Neuropsychology and Psychopharmacology, Faculty of Psychology and Neuroscience, Maastricht University, Maastricht, The Netherlands; Wake Forest School of Medicine, United States of America

## Abstract

The default-mode network (DMN), which comprises medial frontal, temporal and parietal regions, is part of the brain’s intrinsic organization. The serotonergic (5-HT) neurotransmitter system projects to DMN regions from midbrain efferents, and manipulation of this system could thus reveal insights into the neurobiological mechanisms of DMN functioning. Here, we investigate intrinsic functional connectivity of the DMN as a function of activity of the serotonergic system, through the administration of the selective serotonin reuptake inhibitor (SSRI) escitalopram. We quantified DMN functional connectivity using an approach based on dual-regression. Specifically, we decomposed group data of a subset of the functional time series using spatial independent component analysis, and projected the group spatial modes to the same and an independent resting state time series of individual participants. We found no effects of escitalopram on global functional connectivity of the DMN at the map-level; that is, escitalopram did not alter the global functional architecture of the DMN. However, we found that escitalopram decreased DMN regional pairwise connectivity, which included anterior and posterior cingulate cortex, hippocampal complex and lateral parietal regions. Further, regional DMN connectivity covaried with alertness ratings across participants. Our findings show that escitalopram altered intrinsic regional DMN connectivity, which suggests that the serotonergic system plays an important role in DMN connectivity and its contribution to cognition. Pharmacological challenge designs may be a useful addition to resting-state functional MRI to investigate intrinsic brain functional organization.

## Introduction

The brain’s functional organization includes a network of medial frontal and parietal cortex and hippocampal areas [1]–[4]. This network has been termed the “default mode” network (DMN) [5] because it typically shows increased metabolic activity during resting baseline compared to periods of goal-directed cognitive behaviour. The DMN is thought to play a role in a number of cognitive and affective behaviours, including social and self-referential perception [6]–[8], internal mental representation [9] and memory [4], [10]–[13]. Furthermore, the DMN may be involved in the neuropathology of a number of clinical disorders. For example, memory impairments in Alzheimer’s Disease (AD) may be associated with impaired hippocampal connectivity with the remainder of the DMN [14]–[16]. Also, impaired medial frontal, parietal or temporal connectivity may respectively contribute to affective processing deficits in major depression (MD) [17], [18], and psychotic symptoms in schizophrenia [19], [20].

The DMN has also been investigated in many functional magnetic resonance imaging (fMRI) studies that measured intrinsic brain activity, during resting-states in which participants refrained from specific task performance [1]–[3], [21], [22]. The fMRI signal measured during such resting states are likely to have a neurophysiological source [23]–[25], and may influence or facilitate on-line goal-directed cognitive performance [10], [26]–[28]. In this sense, resting-state measurements provide a simple opportunity to study the brain’s functional organization at relatively low cognitive demands for the patients, which makes it an appealing paradigm for clinical research.

However, a more detailed neurobiological understanding of DMN functionality is currently lacking. One possible method to investigate the DMN in humans is to measure the DMN’s intrinsic organization as a function of the activity of neurotransmitter systems. Previously, fMRI has been used to measure brain activity after administration of pharmacological agents that are used to manipulate the activity of particular neurotransmitter systems, compared to placebo (pharmacological fMRI or ph-fMRI). These ph-fMRI studies showed regional differential responses of brain activity after administration of pharmacological agents, compared to placebo [29], [30], as well as regionally specific interactions between agent and task performance [31]–[34]. Previously, we showed that the selective serotonin reuptake inhibitor (SSRI) escitalopram decreased brain activity in thalamic and medial and lateral prefrontal cortical regions, compared to placebo, during the execution of a vigilance task [34]. Escitalopram did not change behavioral performance, but did show a significant decrease in self-reported alertness.

Recently, pharmacological challenge has also been conducted in resting state fMRI measurements in healthy participants. So far, these studies provided little or no evidence of changes in DMN connectivity after administration of alcohol [35], [36] or morphine [36], compared to placebo. However, one study reported that the dopamine agonist L-Dopa decreased functional connectivity of medial DMN regions [37]. Carhart et al. recently showed decreased functional coupling between medial frontal and parietal regions of the DMN after intake of psilocybin [38], a psychedelic compound, which could be associated with increased serotonergic activity [39]. Finally, Northoff and colleagues reported that GABA concentrations in ventromedial prefrontal cortex predicted amount of deactivations in this region of cortex [40]. These findings, thus, indicate that DMN functionality could depend on the interaction between multiple neurotransmitter systems, and that resting-state ph-fMRI may be very suitable to probe this mechanism.

In the current study, we analyzed the effects of the SSRI escitalopram on the intrinsic functional connectivity of the DMN. The serotonergic (5-hydroxytryptamine, 5-HT) neurotransmitter system may play a role in the intrinsic functional dynamics of the DMN for a number of reasons. Ascending serotonergic pathways from the midbrain raphe nucleus project onto many cortical and subcortical limbic system areas, including hippocampal structures, amygdala and cingulate cortex regions, which are part of the DMN functional architecture [1], [2], [5]. Serotonin may play an important role in cognitive functioning in healthy participants [41], [42], such as modulating levels of alertness or sustained attention [43], or long-term memory [44], [45]. Furthermore, pharmacological brain imaging studies showed that administration of serotonergic agents to healthy participants altered brain activity in cingulate areas [29], [30] and hippocampus [29]. Specifically, escitalopram has been found to decrease brain activity in medial cortical and hippocampal areas during task executions [31], [33], [34]. These changes in brain activity are very likely to impact the functional integration of these brain areas within the DMN, which may in turn affect cognitive processing and contribute to pathological manifestations. For example, serotonin dysfunctions have been associated with symptomatology and cognitive impairments in MD [43] [46], as well as in AD [46] and schizophrenia [47]. In MD, selective serotonin reuptake inhibitors (SSRIs), which increase 5-HT synaptic availability or functioning, alleviate affective symptoms and improve memory performance and other cognitive functions (see [43] for review).

We previously obtained two resting-state timeseries of healthy individuals as part of a placebo-controlled, within-subject ph-fMRI study that investigated the effects of escitalopram on the neural correlates of vigilance [34]. We hypothesized that escitalopram, compared to placebo, decreased intrinsic functional connectivity of the DMN. We tested the hypothesis at the level of global connectivity (i.e., at the map-level), and at the level of local (regional) connectivity, as these two levels of analysis may provide related but distinct information about functional network dynamics [48], [49]. We used a dual regression approach [36], [49], [50], in which we first quantified intrinsic DMN functional connectivity during placebo and drug conditions of the first resting-state using group spatial independent component analysis (sICA) [51], [52], and then dual-regressed the spatial modes onto the timeseries of the first and the second resting state segment of individual participants. The effect of escitalopram on global DMN connectivity was assessed using a voxel-by-voxel analysis, and the effect on local connectivity using a pairwise region-of-interest approach.

## Methods

### Subjects

Ten healthy volunteers (5 females), mean age (se) 26.3 (2.46) were recruited. Recruited participants were free of a history of previous use of antidepressant medication (recruitment procedures and in- and exclusion criteria are described elsewhere [34]). The study was approved by the medical ethics committee of Maastricht University and the Maastricht Academic Hospital’s Board of Directors, and was carried out in accordance with the World Medical Association’s *Declaration of Helsinki* (Edinburgh, 2000). Written informed consent was obtained from each volunteer prior to participation in the study.

### Design and Treatment

The resting state measurements were obtained as part of a previous study that measured the effects of escitalopram on vigilance [34]. Here, we briefly reiterate the experimental design and pharmacological parameters. A more elaborate description can be found in our previous text.

The study was conducted according to a double-blind, placebo controlled, 2-way cross-over design. Complete balancing of the treatments led to two treatment orders that were randomly assigned to the participants. Treatments consisted of escitalopram (20 mg) and placebo administered at 2 different test days separated by a wash-out period of at least 7 days.

On the days of measurement, participants arrived at 9.00 a.m. at the laboratory, filled out an informed consent concerning scanning procedures, received a standard breakfast and completed a sleep quality questionnaire. They received the treatment capsule containing either escitalopram or placebo at 9.30 a.m. Oral administration of escitalopram reaches the maximum concentration in blood (Cmax) within 3–4 hours, and has a half-life elimination of 27–32 hours [53]. Participants were then seated for the next hours in a secluded waiting room in order to wait for escitalopram to reach Cmax. At noon participants received a standard light lunch, followed by a self-report assessment of alertness, contentedness and calmness [54] (visual analogue scales [VAS]; range, 0–100; 0 = low, 100 = high). As reported previously [34], participants reported significantly lower Alertness ratings after escitalopram administration (Alertness = 67.0) compared to placebo (Alertness = 81.4; T(9) = −4.6, P = 0.001). Ratings on contentedness and calmness did not significantly differ between the two drug conditions. Scanning and testing took place at 13.30 p.m., i.e. 4 hrs after drug intake, till 14.15 p.m. Participants were not allowed to consume alcohol 24 hours prior to testing and caffeine-containing beverages 4 hours prior to the start of the measurement day.

### FMRI Data Acquisition

Measurements were acquired using a 3T Siemens Allegra MR scanner. A T1-weighted anatomical scan was acquired for each participant using a 3D modified driven equilibrium fourier transform (MDEFT) sequence (176 slices; in-plane resolution, 1 mm^∧^2). A T2*-weighted functional measurement was acquired using an echo-planar image (EPI) pulse sequence (1,316 whole-brain volumes; 32 slices; slice thickness, 3.5 mm; no slice gap; flip angle 90°; TR/TE, 2,000/30 msec; in-plane resolution, 3.5×3.5 mm^∧^2, matrix size, 64×64) and interleaved slice sampling. The complete timeseries comprised a first resting state measurement (volumes 1–210), followed by the vigilance task (211–1106), and ended with a second resting state measurement (1107–1316). During the resting measurement, participants fixated their gaze on a fixation cross, and no additional stimulus or task was presented.

### Data Preprocessing

The first two volumes of each complete time series were discarded because of saturation effects. Preprocessing of the functional images was done using BrainVoyager QX version 1.6 [55], and included slice time correction, head motion correction, spatial smoothing (Gaussian kernel with full-width-at-half-maximum of 6 mm), and linear trend removal of time courses and high pass temporal filtering of 5 cycles per time course (∼ 0.0019 Hz). Individual anatomical datasets were spatially normalized to a standardized 3-dimensional (3D) space [56]. Individual functional images were co-registered and normalized to the anatomical data, and resampled to a voxel size of 3×3×3 mm^∧^3. The standardized anatomical images of the participants were averaged and a group-based volume mask was created that tagged voxels belonging to cerebral and cerebellar matter (selecting 50,381 voxels, ≈ 47% of total volume), and excluded voxels belonging to ventricular space or tissue outside of the brain. For the first and second resting state segments, RS1 and RS2, we dropped the respective last and first four volumes in order to prevent any effects of task on- or offset. Thus, each resting-state segment included 204 timepoints.

### Functional Connectivity Analysis

Intrinsic functional connectivity was estimated for the two resting-state segments of the time series (RS1 and RS2). We used spatial independent component analysis (sICA) to decompose the RS1 timeseries of all participants, but separately for escitalopram and placebo runs, into a set of 40 spatial modes. For this analysis, RS1 timeseries were normalized and concatenated across time, resulting in an aggregated data matrix of 2,040 volumes ( = 10 participants×204 volumes) by 50,381 voxels for the escitalopram (i.e., RS1E) and placebo (RS1P) runs. FastICA [57] was used to spatially decompose each aggregate data matrix into 40 independent components using the symmetric decomposition option. Initial dimension reduction was performed using principal component analysis (PCA). Details of the spatial ICA decomposition in fMRI are described elsewhere [22], [51], [52], [58], [59]. We used a spatial template of the posterior cingulate cortex (PCC) from an independent study [22] (center of mass [x, y, z] = −1, −47, 24; size = 13,319 mm^∧^3) to label and select one DMN functional connectivity map in each decomposition according to the highest absolute correlation with the spatial template (RS1E max(|r|) = 0.43; RS1P max(|r| ) = 0.42).

Following the dual-regression analysis scheme [36], [60], [61], we first spatially regressed the (Z-normalized) spatial modes of the RS1E and RS1P decompositions onto the RS1 and RS2 timeseries of the respective drug condition. Thus, the spatial modes were applied to the data from which they were estimated (i.e., RS1), and to a second dataset of the same participants (RS2). This step resulted in a set of temporal profiles of the spatial modes. In the second step, we temporally regressed the temporal profiles onto the complete functional runs.

We then analysed the effects of escitalopram on DMN connectivity at the map-level, which assesses global (multivariate) connectivity, and at the regional-level, which assesses local (pairwise) connectivity. The two levels of analysis can provide related but distinct information about the effects of escitalopram on network connectivity [48], [49]. To assess map-level effects of escitalopram we investigated the voxel-by-voxel results of the DMN temporal profiles, comparing DMN connectivity between the two drug conditions. To assess regional effects of escitalopram on DMN connectivity, we followed a region-of-interest (ROI) approach, in which ROIs were obtained from a one-sample t-test map of the functional connectivity values across all conditions and timeseries. From the ensuing ROIs we sampled timeseries from DMN regions and removed effects of non-DMN temporal profiles (obtained from the first dual-regression step), and a number of fMRI covariates [3], [62]–[64] including head movement, fMRI signal from the ventricles and from white matter, and signal oscillations at a frequency above 0.1 Hz (using pairs of discrete sines and cosines). The corrected ROI timeseries were then segmented according to the RS1 and RS2 time windows, Z-normalized and pair-wise correlated using the Pearson correlation coefficient. Correlation coefficients *r* were then transformed to normality using Fisher’s Z normalization for further analysis using repeated measures ANOVA (RMANOVA) with within-subject factors Drug (Escitalopram, Placebo) and Time (RS1, RS2), and their Drug x Time interaction term. Pairwise ROI connections that showed a significant effect on at least one of these factors were considered for post-hoc analysis, which included paired-sample t-tests (df = 9). Functional connectivity estimation and statistical analysis of the results were performed in Matlab (MathWork, Inc.), in which we used an adapted version of the RMANOVA implementation written by Trujillo-Ortiz et al. [65]. Correction for multiple pairwise comparisons was performed using a false-discovery rate (FDR) of q = 0.05 [66].

To define DMN ROIs for the regional connectivity analysis, we averaged the spatial maps from the second dual-regression step across the two resting states and the two drug conditions, and calculated a mass-univariate one-sample t-test map, which was thresholded using q(FDR) = 0.05 and minimum cluster size of 270 mm^∧^3, corresponding to a cluster-level threshold alpha = 0.05, as estimated by a simulation procedure (1,000 Monte Carlo simulations) of the statistical map that is based on its estimated spatial smoothness [55], [67]. This procedure resulted in seven ROIs, which included anterior cingulate cortex/ventromedial prefrontal cortex (ACC), posterior cingulate cortex/precuneus (PCC), left and right hippocampal and parahippocampal complex (LPHC and RPHC), left and right inferior parietal cortex/posterior part of the superior temporal gyrus (LIPC and RIPC), and left middle frontal gyrus (LMFG). Pairwise correlations resulted in 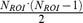  = 21 unique correlations per resting state, drug condition and participant. **Table 1** lists the ROI sizes in mm^∧^3 and Talairach coordinates.

**Table 1:** ROI details. ROI, region-of-interest; k, voxel cluster size in mm^∧^3; x,y,z, Talairach coordinates in mm.

ROI	k	x	y	z
PCC	41500	0	−51	25
RIPC	9338	44	−59	22
LIPC	6136	−44	−59	20
RPHC	1547	21	−14	−18
ACC	1287	1	53	6
LPHC	846	−22	−11	−18
RMFG	649	23	21	48

## Results

**Figure 1:**
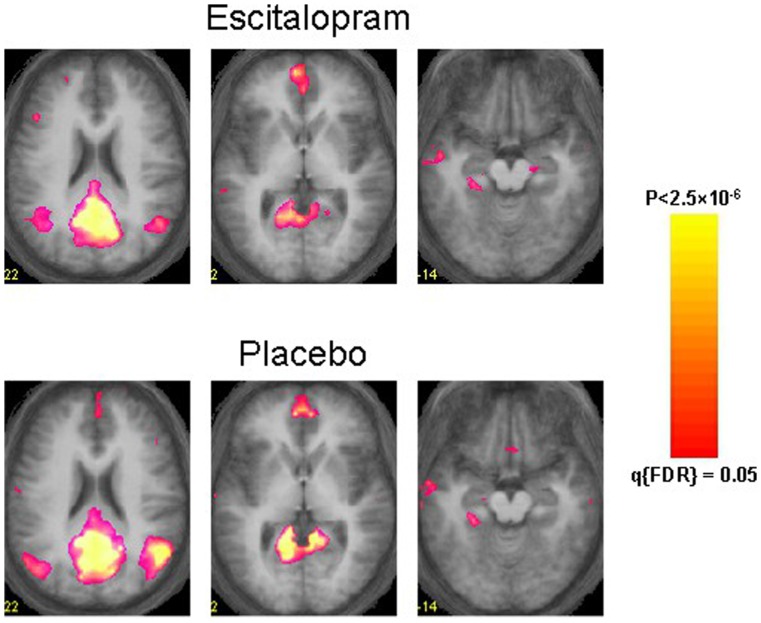
Default-mode networks after escitalopram (upper row) and placebo administration (bottom row). The figure shows three transverse slices (Talairach-Z coordinates 22, 2 and −14) of ICA-derived functional connectivity maps of the DMN superimposed on the anatomical average of the participants. The left hemisphere is depicted on the left side of each image.

The effect of escitalopram was first assessed at the map level using a mass-univariate, voxel-by-voxel RMANOVA of the dual-regression estimates for functional connectivity. **Figure 1** shows the spatial DMN maps obtained from the aggregated sICA of the RS1 segment of the escitalopram (**Fig. 1** **, upper row**) and placebo runs (**Fig. 1** **, bottom row**). The sICA results were used as input to the dual-regression analysis to estimate global functional connectivity of the DMN for the escitalopram and placebo functional timeseries of RS1 and RS2. The ensuing results were analysed using a voxel-by-voxel RMANOVA, and results were thresholded using q(FDR) = 0.05 and a minimum cluster size of 270 mm^∧^3. The analysis yielded no significant effects of Drug, Time or the Drug x Time interaction effect.

**Figure 2:**
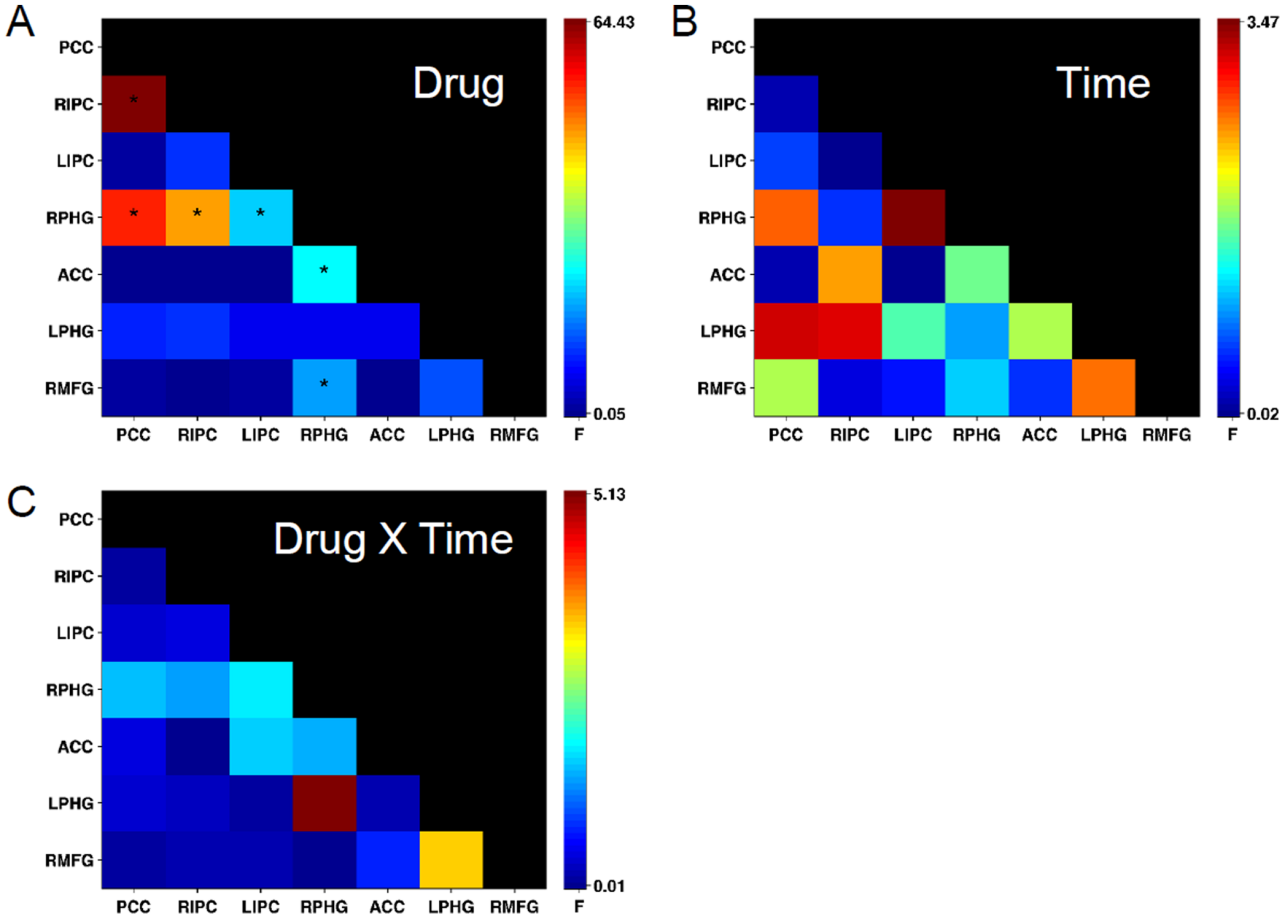
ROI-based RMANOVA results. **Each panel depicts the color-coded RMANOVA F-values of the pairwise ROI functional connections for factors Drug (A), Time (B) and Drug x Time (C).** Repetitive values in the ROI connections matrix are replaced with black. Degrees of freedom for plotted F-values are 1 and 9 in all cases. Asterisks mark ROI connections with significant results at q(FDR) = 0.05.

**Figure 3:**
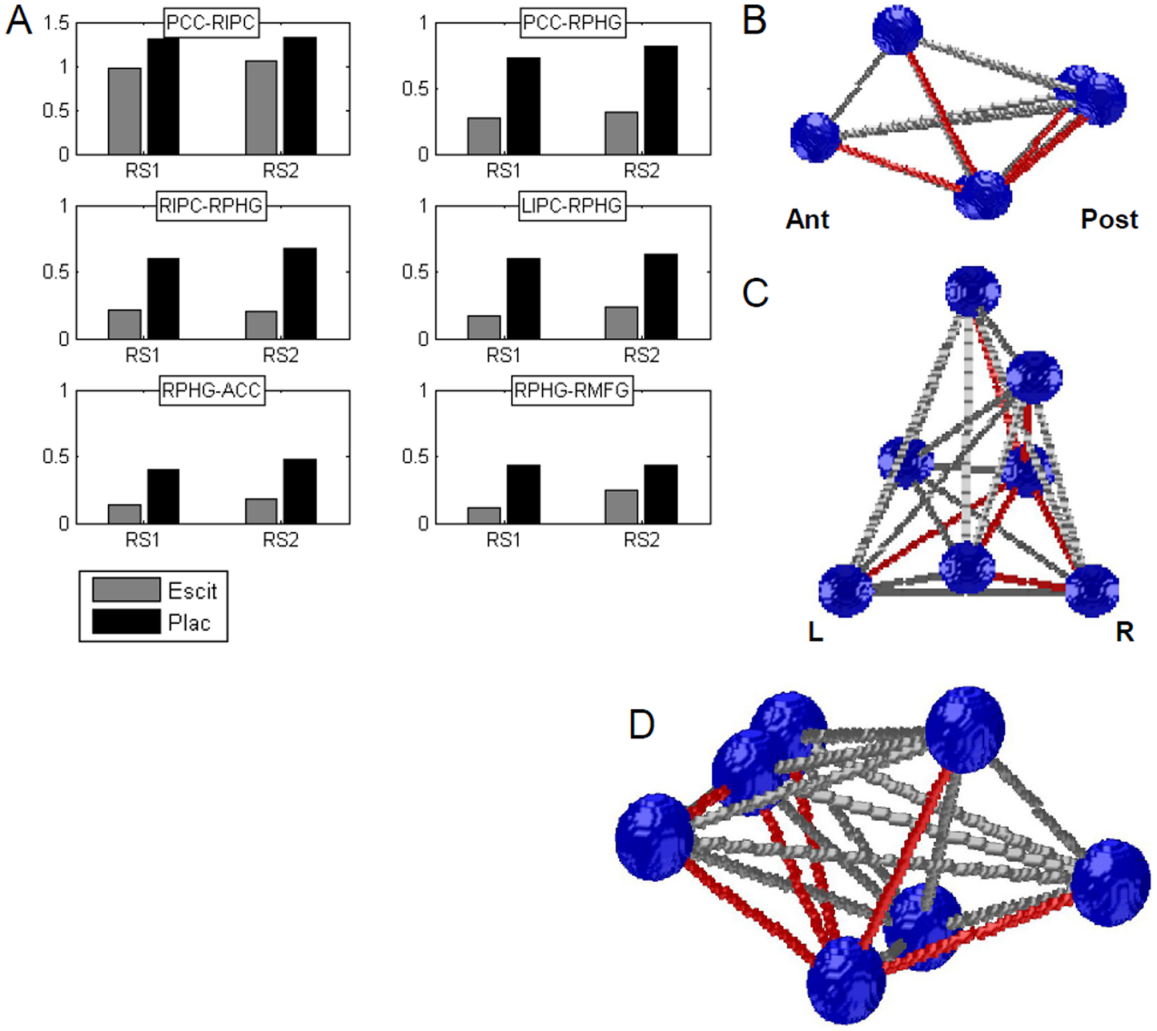
Escitalopram-induced changes in DMN functional connectivity. A: Bars show means of functional connectivity values (Fisher’s Z normalized correlation coefficients) as a function of drug condition, and for each resting-state segment, for each of the six connections that were found significant after RMANOVA testing. Grey bars represent connectivity after Escitalopram administration, black bars after Placebo. B-D: Schematic 3D display of the DMN ROIs and their pairwise functional connections. ROIs are displayed as blue orbs (radius = 10 mm), significant connections are displayed as red beams connecting the orbs, non-significant connections are shown as grey beams. B: Lateral view (sagittal; left side is anterior). C: Top view (transverse; left side is left). D: Tilted side view.

The effect of escitalopram on regional connectivity between the seven DMN ROIs was assessed using a pairwise ROI approach. ROIs included ACC, PCC, LPHC, RPHC, LIPC and RIPC (see **Table 1** for further details). **Figure 2** shows the color-coded F-values for the RMANOVA within-subject factors of Drug (**Fig. 2A**) and Time (**Fig. 2B**), and the Drug x Time interaction term (**Fig. 2C**). After FDR correction for multiple comparisons of pairwise ROI correlations, seven functional connections showed a significant effect of Drug (marked by asterisk in **Fig. 2A**). The pairwise connections included PCC-RIPC, PCC-RPHC, RIPC-RPHC, LIPC-RPHC, ACC-RPHC and RPHC-RMFG. Functional connections showed no significant effects of Time or of the interaction between Drug and Time, which suggests that escitalopram-related changes in functional connectivity between DMN ROIs was similar for both resting states RS1 and RS2. Post-hoc paired-sample t-tests of the seven connections showed that escitalopram reduced pairwise functional connectivity strength, compared to Placebo, in both RS1 and RS2. Barplots of **Figure 3A** show the respective means of the connectivity values for the escitalopram (grey bars) and Placebo condition (black bars), for both resting states RS1 and RS2, and **Table 2** lists the statistical values of the comparisons. We did not observe any significant effect of Time or the Drug x Time interaction, which indicated that the DMN connectivity between the two resting-state segments did not differ.

**Table 2:** ROI connection details.

	RS1				RS2			
Conn	Escit	Plac	T	P	Escit	Plac	T	P
PCC-RIPC	.98	1.30	−3.7	.005	1.06	1.32	−4.0	.003
PCC-RPHC	.27	.73	−4.7	.001	.32	.82	−7.6	<.001
RIPC-RPHC	.21	.60	−3.4	.008	.20	.68	−8.5	<.001
LIPC-RPHC	.17	.60	−4.2	.002	.24	.63	−4.6	.001
RPHC-ACC	.14	.40	−2.5	.037	.18	.48	−6.0	<.001
RPHC-RMFG	.12	.43	−3.6	.006	.25	.43	−2.2	0.058

For each significant pairwise connection (Conn) the average connectivity values after Escitalopram (Escit) and Placebo (Plac) administration are shown, together with the paired-sample T-test statistics (degrees of freedom = 9), for both resting states RS1 and RS2. Abbreviations, see main text.

**Figures 3B–D** show a schematic 3D overview of the pairwise functional architecture of the DMN (sagittal view in **Fig. 3B**, transverse view in **Fig. 3C** and tilted sagittal view in **Fig. 3D**), with the DMN regions presented as blue orbs (for visual display purposes, orbs of 10 mm radius replaced actual ROIs at their center coordinates) and the functional connections as grey or red beams. Functional connections that showed a significant drug-related difference are marked in red. Note that the connections do not imply anatomical connectivity, or directed or conditional influences between regions.

### Regional DMN Connectivity and Alertness

**Figure 4:**
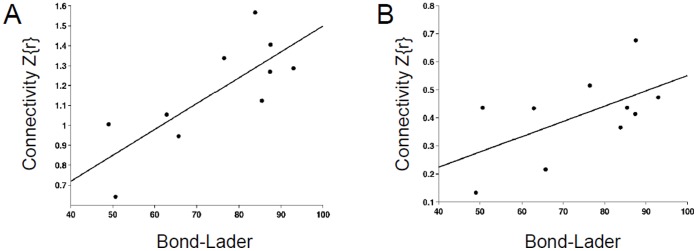
Scatterplots. Shown are the scatterplots of functional connectivity between PCC and RIPC (A), and between LIPC and RPHC (B) as a function of alertness ratings of the Bond and Lader VAS scale. Each data point represents one participant. A regression line (least squares fit) is drawn through the data points in each plot. Functional connectivity and VAS rating values are the average of the Escitalopram and Placebo conditions.

To investigate if regional functional connectivity contributed to self-reported alertness of the Bond and Lader VAS scale, we correlated the alertness ratings with the functional connectivity values of the seven connections. Alertness ratings and connectivity coefficients of escitalopram and placebo were averaged for each participant before correlation analysis. Decreased alertness correlated significantly with decreased posterior cingulate-parietal connectivity (PCC-RIPC: r = 0.78, P = 0.007; see **Fig. 4A**), and marginally with decreased hippocampal-parietal connectivity (LIPC-RHPC: r = 0.58, P = 0.08; see **Fig. 4B**). Thus, self-report level of alertness covaried with intrinsic DMN connectivity.

## Discussion

In this study we investigated if the SSRI escitalopram affected functional connectivity of the DMN during resting-state fMRI measurements. In both pharmacological conditions the DMN contained areas that have been reported in previous resting-state studies [1], [2], [4], [5]. Escitalopram showed decreased regional pairwise connectivity between frontal, parietal and temporal regions, including hippocampus, parietal cortex and ventromedial cortex. These effects were found in two resting-state segments of the same individuals, suggesting that these effects are reliable and stable over time. We interpret these findings as evidence that escitalopram altered intrinsic DMN functional connectivity through the modulation of the functionality of the serotonergic system in the brain.

Our results fit with previous reports of regional changes in brain activity after serotonergic challenge. In a pharmacological challenge PET study, Mann et al. [30] administered the SSRI fenfluramine to healthy participants and found increased and decreased activity in respectively the ACC and PCC, compared to placebo. Geday et al. [68] showed in a pharmacological PET study decreased ACC activity after citalopram administration. Further, several fMRI studies showed decreased task-related activity in medial cortical, hippocampal, thalamic and cerebellar regions after SSRI administration [33], [34], [69], [70]. In contrast, McKie et al. [29] used citalopram in a pharmacological challenge fMRI study and found increased activity in the subgenual part of the ACC, medial thalamus and hippocampus. These changes in SSRI-induced regional activity changes suggest that functional coupling between these and other areas may be reduced after serotonergic challenge. A recent report of decreased functional coupling between ACC and PCC after intake of the psychedelic psilocybin [38] lends further support to this suggestion, because the effects of psychedelic compounds may be initiated by stimulation of 5-HT receptors [39], [71].

Findings from receptor mapping studies provide further support for the suggestion that escitalopram-related changes to DMN functional connectivity were based on altered serotonergic functioning. Receptor mapping studies in rodent and non-human primates showed that serotonergic afferents project to multiple regions of the limbic system, which include anterior and posterior portions of the cingulate cortex [72], [73] and hippocampus [42], [74]. A rat ph-fMRI study showed increased functional coupling amongst sub-cortical regions that were part of serotonin pathways, including raphe nuclei, hippocampus and thalamus in the rat brain after fluoxetine challenge [75]. Finally, neurophysiological studies in animals show that the results of our and other neuroimaging studies are likely associated with changes in metabolic demands as a consequence of altered serotonin levels, rather than with vasodynamics unrelated to neurophysiology [30], [75], [76]. Of potential relevance here are reports that the neurophysiological basis of the fMRI signal may capitalize more on synaptic activity, rather than on neuronal spike or firing rates [77]–[79], which could indicate that serotonergic effects on neurophysiology occur most strongly at synaptic connection sites.

Our results also show that escitalopram-induced changes in DMN functional organization may have contributed to decreased alertness or vigilance. This finding fits with the DMN’s putative involvement in ongoing monitoring of internal representations when the brain is not engaged in a stimulus-driven task [80], [81]. This association may be relevant for task performance, in which changes in vigilance or sustained attention can influence task-related processing. Reduced DMN deactivation during vigilance tasks may indicate that the DMN is involved in reallocating processing resources away from the task at hand [9], [82], [83]. Furthermore, several studies have shown that administration of SSRIs may impair vigilance in healthy participants [43], [84], [85], which further supports our interpretation that escitalopram altered DMN functionality through altered serotonergic functioning.

Our findings could be relevant for understanding the neurobiological mechanism of the DMN’s contribution to cognition. For example, there is growing evidence that posterior regions of the DMN are involved in (episodic) memory processing [4], [10], [12], [86], [87]. Previous studies showed that decreased serotonin levels may impair long-term memory performance in healthy participants [44], [45], and increased 5-HT levels may improve [88] as well as impair memory performance [84], [89]. A recent placebo-controlled ph-fMRI study in which 3,4-methylenedioxy-methamphetamine (MDMA) was administered to healthy participants showed reduced deactivation in inferior parietal areas, compared to placebo, which correlated with increased prospective memory failure [90]. These authors concluded that reduced activation in inferior parietal cortex may have resulted from increased serotonergic availability after acute MDMA administration, thereby impairing memory performance.

Our study focused primarily on the functional connectivity of the DMN, but it is possible that escitalopram affected other resting-state networks as well. The intrinsic functional architecture of the human brain comprises multiple neural networks [91], [92]. Serotonergic afferents project to many different cortical and subcortical areas beyond DMN regions [42], [93]. Serotonergic activity is important for synaptic plasticity and functional organization in the mammalian primary visual cortex [79], [94], [95], and may play a role in reorganization of neural networks of the motor system [69], [96]. At the same time, ph-fMRI studies provide little evidence for serotonergic effects in lateral prefrontal and posterior parietal cortex [97], which are commonly associated with executive control and attentional task performance [3], [98]. Thus, it would be of interest to investigate in a more exploratory fashion which resting-state networks escitalopram affects. However, this approach is beyond the scope of the current paper.

A few issues deserve consideration. The small sample size, comparable to previous ph-fMRI studies [97], could have under-powered the map-level analysis. However, our findings indicate that a regional analysis may be more appropriate than a voxel-by-voxel analysis in possible low-power cases. An important methodological consideration in resting-state fMRI is the possible confounding effect of physiological responses, such as cardiac or respiratory rate, to measurements of intrinsic brain activity [62], [63], [99], which may be further increased in pharmacological challenge studies. Several studies did not find significant differences in heart rate after escitalopram administration, compared to placebo [100], [101], although small decreases have been reported [102]. We aimed to minimize possible influences to the functional connectivity estimates in several ways. Spatial ICA can reliably separate functional connectivity maps of putative neurophysiological sources from physiological responses**,** such as respiratory or pulse rate, and other signal artifacts [62], [103]–[105]. We also removed signals from ventricular and white matter areas from the functional connectivity estimates [62], and restricted our analysis to signal fluctuations occurring below 0.1 Hz. A second methodological issue concerns the application of dual-regression analysis to different segments of the data. Using the sICA solution of an aggregated dataset in the dual-regression approach indirectly imposes a desirable data reduction step that is similar to group-based PCA [50]. However, this also introduces circularity or re-usage of the data in the analysis pipeline. In our study, we used the sICA solution of one segment of the data to explain the same and another, independent segment of the functional timeseries, and showed that the functional connectivity results did not differ between the two segments. Thirdly, the two resting-state measurements were extracted from a larger dataset in which participants performed a vigilance task in-between the resting states. There are some indications that task performance may alter the functional architecture of *post-task* resting-state networks [26], [106]–[108]. However, we found no evidence for an effect of the vigilance task on global or local intrinsic DMN connectivity, indicating that the task did not bias our main results. Finally, we did not obtain resting-state baseline measurements prior to experimental manipulation.

In conclusion, escitalopram administration to healthy participants decreased functional connectivity of medial frontal, parietal and hippocampal regions within the DMN. These regional changes appear in line with reported behavioral and functional imaging results after administration of serotonergic agents. We propose that resting-state ph-fMRI is a valuable neuroimaging tool to further investigate the intrinsic functional architecture of the brain in healthy and clinical populations.
